# The etiological roles of miRNAs, lncRNAs, and circRNAs in neuropathic pain: A narrative review

**DOI:** 10.1002/jcla.24592

**Published:** 2022-07-09

**Authors:** Ming Jiang, Yelong Wang, Jing Wang, Shanwu Feng, Xian Wang

**Affiliations:** ^1^ Department of Anesthesiology and Pain Medicine, Nanjing Maternity and Child Health Care Hospital Women's Hospital of Nanjing Medical University Nanjing China; ^2^ Department of Anesthesiology Gaochun People's Hospital Nanjing China

**Keywords:** circRNAs, lncRNAs, mechanism study, miRNAs, neuropathic pain

## Abstract

**Background:**

Non‐coding RNAs (ncRNAs) are involved in neuropathic pain development. Herein, we systematically searched for neuropathic pain‐related ncRNAs expression changes, including microRNAs (miRNAs), long non‐coding RNAs (lncRNAs), and circular non‐coding RNAs (circRNAs).

**Methods:**

We searched two databases, PubMed and GeenMedical, for relevant studies.

**Results:**

Peripheral nerve injury or noxious stimuli can induce extensive changes in the expression of ncRNAs. For example, higher serum miR‐132‐3p, ‐146b‐5p, and ‐384 was observed in neuropathic pain patients. Either sciatic nerve ligation, dorsal root ganglion (DRG) transaction, or ventral root transection (VRT) could upregulate miR‐21 and miR‐31 while downregulating miR‐668 and miR‐672 in the injured DRG. lncRNAs, such as early growth response 2‐antisense‐RNA (Egr2‐AS‐RNA) and Kcna2‐AS‐RNA, were upregulated in Schwann cells and inflicted DRG after nerve injury, respectively. Dysregulated circRNA homeodomain‐interacting protein kinase 3 (circHIPK3) in serum and the DRG, abnormally expressed lncRNAs X‐inactive specific transcript (XIST), nuclear enriched abundant transcript 1 (NEAT1), small nucleolar RNA host gene 1 (SNHG1), as well as ciRS‐7, zinc finger protein 609 (cirZNF609), circ_0005075, and circAnks1a in the spinal cord were suggested to participate in neuropathic pain development. Dysregulated miRNAs contribute to neuropathic pain via neuroinflammation, autophagy, abnormal ion channel expression, regulating pain‐related mediators, protein kinases, structural proteins, neurotransmission excitatory–inhibitory imbalances, or exosome miRNA‐mediated neuron–glia communication. In addition, lncRNAs and circRNAs are essential in neuropathic pain by acting as antisense RNA and miRNA sponges, epigenetically regulating pain‐related molecules expression, or modulating miRNA processing.

**Conclusions:**

Numerous dysregulated ncRNAs have been suggested to participate in neuropathic pain development. However, there is much work to be done before ncRNA‐based analgesics can be clinically used for various reasons such as conservation among species, proper delivery, stability, and off‐target effects.

## Graphical abstract

Mechanism study of dysregulated microRNAs (miRNAs), long non‐coding RNAs (lncRNAs), and circular non‐coding RNAs (circRNAs) for neuropathic pain development.

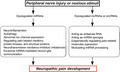

## 1 INTRODUCTION

According to the International Association for the Study of Pain, neuropathic pain is the most severe chronic pain condition triggered by a lesion or disease of the somatosensory system. It is characteristic of hyperalgesia, allodynia, or spontaneous pain.^1^ Neuropathic pain can have peripheral and central origins, with the former including neuropathic pain after peripheral nerve injury, trigeminal neuralgia, postherpetic neuralgia, painful radiculopathy, and painful polyneuropathy. In contrast, central neuropathic pain includes neuropathic pain after spinal cord or brain injury, multiple sclerosis, and chronic central post‐stroke pain. Approximately 7%–10% of the general population will experience neuropathic pain, with the majority not having satisfactory pain relief with current therapies, leading to great suffering for individuals and enormous economic and social burdens.^2^

The exact molecular mechanisms underlying neuropathic pain remain unclear, and elucidating them is crucial for developing mechanism‐based treatment strategies. One proposed mechanism involves altered gene or protein expression along the pain processing pathways. Therefore, understanding how genes or proteins are dysregulated may help us to find a way to normalize these abnormalities and treat neuropathic pain.

In recent years, accumulating evidence has suggested the essential role of non‐coding RNAs (ncRNAs) in various physiological and pathological procedures, such as embryonic development, inflammation, tumors, and respiratory and cardiovascular diseases.^3^ ncRNAs have no protein‐coding potential, but they can govern gene or protein expression with diverse mechanisms. ncRNAs are extensively distributed in the peripheral and central nervous systems, including pain‐related structures.^4^ Broad abnormal ncRNAs expression is observed following peripheral stimulation. These abnormalities are related to hyperalgesia during chronic pain development. The available data indicate that ncRNAs may be essential for hyperalgesia. In this review, we focus on microRNA (miRNA), long non‐coding RNA (lncRNA), and circular non‐coding RNA (circRNA) expression changes in neuropathic pain. Other types of ncRNAs are seldom reported in neuropathic pain and, thus, are not discussed herein. Notably, we will pay attention to their etiological role in the development of neuropathic pain and the current challenges and considerations for miRNA‐, lncRNA‐, and circRNA‐based therapeutics for neuropathic pain.

## 2 BIOGENESIS AND FUNCTION OF miRNAs, lncRNAs, AND circRNAs

Typically, miRNA production involves three steps: cropping, exporting, and dicing. First, the miRNA gene is transcribed in the nucleus, mainly by RNA polymerase II.^5^ The resulting primary miRNA transcript (pri‐miRNA) is several kilobases (kb) in length, with a specific stem–loop structure that harbors mature miRNAs in the stem. The mature miRNA is ‘cropped’ by Drosha and its interactor DGCR8 (DiGeorge syndrome critical region 8), which cleaves pri‐miRNA at the stem to produce pre‐miRNA,^6^ 60–70 nucleotides (nt) in length with a hairpin structure. Then, exportin‐5 recognizes and exports pre‐miRNA from the nucleus to the cytoplasm.^7^ In the cytoplasm, ribonuclease III (RNAse III), termed Dicer, further cleaves the pre‐miRNA to release double‐stranded miRNA with a length of ~22 nt.^8^ This miRNA is unwound by an unknown helicase or cleaved by Argonaute (Ago) to form the RNA‐induced silencing complex.^9^ One strand in the RNA duplex remains with Ago as a mature miRNA, and the other is degraded. The seed sequence of the miRNA incompletely or entirely combines with the target mRNA sequence, resulting in target mRNAs degradation or transcriptional regulation.^10^

Unlike miRNAs, lncRNAs are mRNAs‐like transcripts ranging in length from 200 nt to 100 kb that lack prominent open reading frames.^11^ The lncRNA cellular mechanism is highly related to their intracellular localization. lncRNAs control chromatin functions, transcription, and RNA processing in the nucleus and affect mRNA stability, translation, and cellular signaling in the cytoplasm.^12^ Compared to lncRNAs, circRNAs are more stable because a single circRNA molecular ends can be covalently linked compared to linear RNA. circRNAs are evolutionarily conserved molecules that are essential in the post‐transcriptional modification of gene expression by acting as miRNAs sponges or interacting with transcription or translational machinery. Numerous lncRNAs and circRNAs are distributed within pain‐related regions and dysregulated after peripheral noxious stimulation. Moreover, functional studies have indicated that miRNAs, lncRNAs, and circRNAs participate in neuropathic pain development by regulating diverse pain‐related genes along the pain processing pathways.

## 3 PERIPHERAL NERVE INJURY OR NOXIOUS STIMULI INDUCE EXTENSIVE miRNAs, lncRNAs, AND circRNAs EXPRESSION CHANGES

### 3.1 miRNAs expression changes

Microarray and deep‐sequencing analyses revealed that nerve injury or noxious stimuli could induce broad changes in miRNA expression in serum or along the pain processing pathways, including the perineal nerve, dorsal root ganglion (DRG), spinal cord, and supraspinal regions. For normal pain signal transmission, nerve injury or noxious stimuli are detected by nociceptors in the DRG or trigeminal ganglion (TG) and then transmitted to upstream neurons in the spinal dorsal horn. Subsequently, nociceptive stimuli are integrated, processed, and further transmitted ascending to specific supraspinal brain regions.

Human studies have identified as many as 1134 differentially expressed (DE) genes in the serum of individuals with or without neuropathic pain after spinal cord injury (SCI).^13^ miR‐204‐5p, ‐519d‐3p, ‐20b‐5p, and ‐6838‐5p might act as promising biomarkers and intervention targets for preventing and therapizing neuropathic pain after SCI. In trigeminal neuralgia individuals, serum miR‐132‐3p, ‐146b‐5p, ‐155‐5p, and ‐384 levels were prominently increased compared with healthy controls.^14^ Patients with painful peripheral neuropathy had higher miR‐21 level in the serum and sural nerve compared with healthy controls. Meanwhile, miR‐155 was reduced in the serum and inflicted lower leg skin.^15^ Animal and human studies showed that miR‐30c‐5p was upregulated in the serum and cerebro spinal fluid (CSF) of sciatic nerve injury rats and neuropathic pain patients with chronic peripheral ischemia. The high expression of miR‐30c‐5p, together with other clinical parameters, might be used to predict neuropathic pain development in patients with chronic peripheral ischemia.^16^ Additionally, an animal study showed that plasma‐derived DE extracellular vesicle (EV) miRNAs regulated processes that are essential for neuropathic pain development. Most DE EV miRNAs for inflammation suppression were downregulated, potentially acting as biomarkers and targets in neuropathic pain treatment.^17^

In addition, numerous DE miRNAs have been identified in the DRG. One week after spared nerve injury (SNI), 33 and 39 miRNAs in the DRG were upregulated and downregulated, respectively, with most DE miRNAs related to axon guidance, focal adhesion, Ras and Wnt signaling pathways.^18^ Furthermore, nerve injury‐induced miRNAs expression was dynamic and time‐dependent,^19^, ^20^ implicating multiple regulatory mechanisms in neuropathic pain initiation and development. Nerve injury redistributes miRNAs from a uniform style within the DRG soma of non‐allodynic animals to preferential localization to peripheral neurons in allodynic animals.^21^ Furthermore, either sciatic nerve ligation (SNL), DRG transaction (DRT), or ventral root transection (VRT) could upregulate miR‐21 and miR‐31 while downregulating miR‐668 and miR‐672 in the injured DRG,^22^ implying that these miRNAs could be therapeutic targets for treating diverse types of neuropathic pain.

The spinal cord dorsal horn relays and modulates pain signals from the peripheral nociceptors to the supraspinal regions. In the spinal cord, numerous miRNA expression changes have been observed in chronic constriction injury (CCI) and diabetic neuropathic pain (DNP) rodents.^23^, ^24^, ^25^, ^26^ Previous research has suggested some miRNAs may be related to neuropathic pain development, for example, miR‐500, ‐221, and ‐21,^25^ thus, acting as potential targets in its treatment.^27^

**FIGURE 1:**
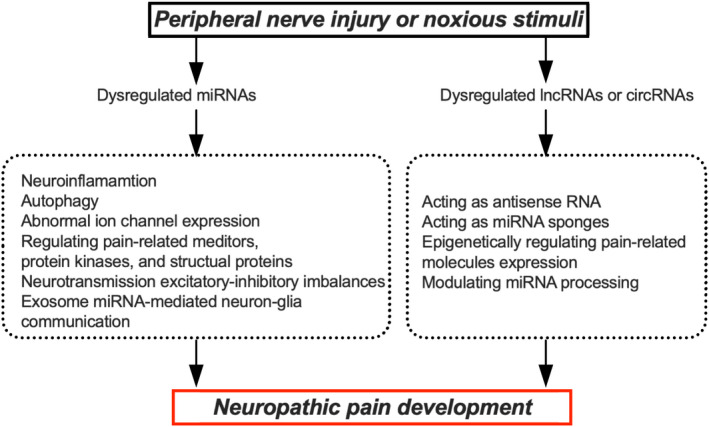
microRNAs (miRNAs) expression change following peripheral nerve injury or noxious stimuli SCI, spinal cord injury; CSF, cerebrol spinal fluid; DE, differential expressed; EV, extravascular vesicle; SNL, spinal nerve ligation; DRG, dorsal root ganglion; SNI, spared nerve injury; CCI, chronic constriction injury; DRT, DRG transection; VRT, ventral root transection

Cytoscape software constructed the miRNA–target gene regulatory network in the supraspinal region, including the nucleus accumbens (NAc), medial prefrontal cortex, and periaqueductal gray, between SNI and sham rats. Finally, four essential DE genes, includingCXCR2, IL12B, TNFSF8, and GRK1, and five miRNAs, including miR‐208a‐5p, −7688‐3p, −344f‐3p, −135b‐3p, and ‐135a‐2‐3p, were identified, indicating their essential roles in neuropathic pain pathogenesis.^28^ Furthermore, in the prelimbic cortex of SNI rats, the DE miRNA–mRNA network pointed to molecules associated with inflammation.^29^ DE miRNAs were also observed in the bilateral hippocampus of CCI rats. However, no significant difference was observed bilaterally in the hippocampus.^30^ (Figure 1).

### 3.2 lncRNAs and circRNAs expression changes

Patients with type 2 DNP had a prominent higher expression of serum lncRNA NONRATT021972 and more severe neuropathic pain symptoms.^31^ LINC01119 and LINC02447 in the peripheral blood of SCI patients were identified in pain pathways that were important for neuropathic pain development.^32^

Microarray analysis revealed that a nerve injury could induce time‐dependent lncRNA expression changes in the sciatic nerve.^33^ lncRNA H19 was persistently upregulated in Schwann cells along the peripheral nerve, proximal and distal to the injured site.^34^ In the DRG, the transcriptomic analysis identified 86 known and 26 novel lncRNAs genes to be DE after spared sciatic nerve injury.^35^ Of these, rno‐Cntnap2 and AC111653.1 were essential in peripheral nerve regeneration and were involved in neuropathic pain.

Next‐generation RNA sequencing showed 134 lncRNAs and 188 circRNAs were prominently changed 14 days after SNI in the spinal cord.^36^ In addition, microarray analysis identified 1481 and 1096 DE lncRNAs and mRNAs, respectively, in the spinal cord dorsal horn of DNP rats. Of these, 289 neighboring and 57 overlapping lncRNA‐mRNA pairs, including ENSMUST00000150952‐Mbp and AK081017‐Usp15, have been suggested to participate in neuropathic pain development.^37^

**FIGURE 2:**
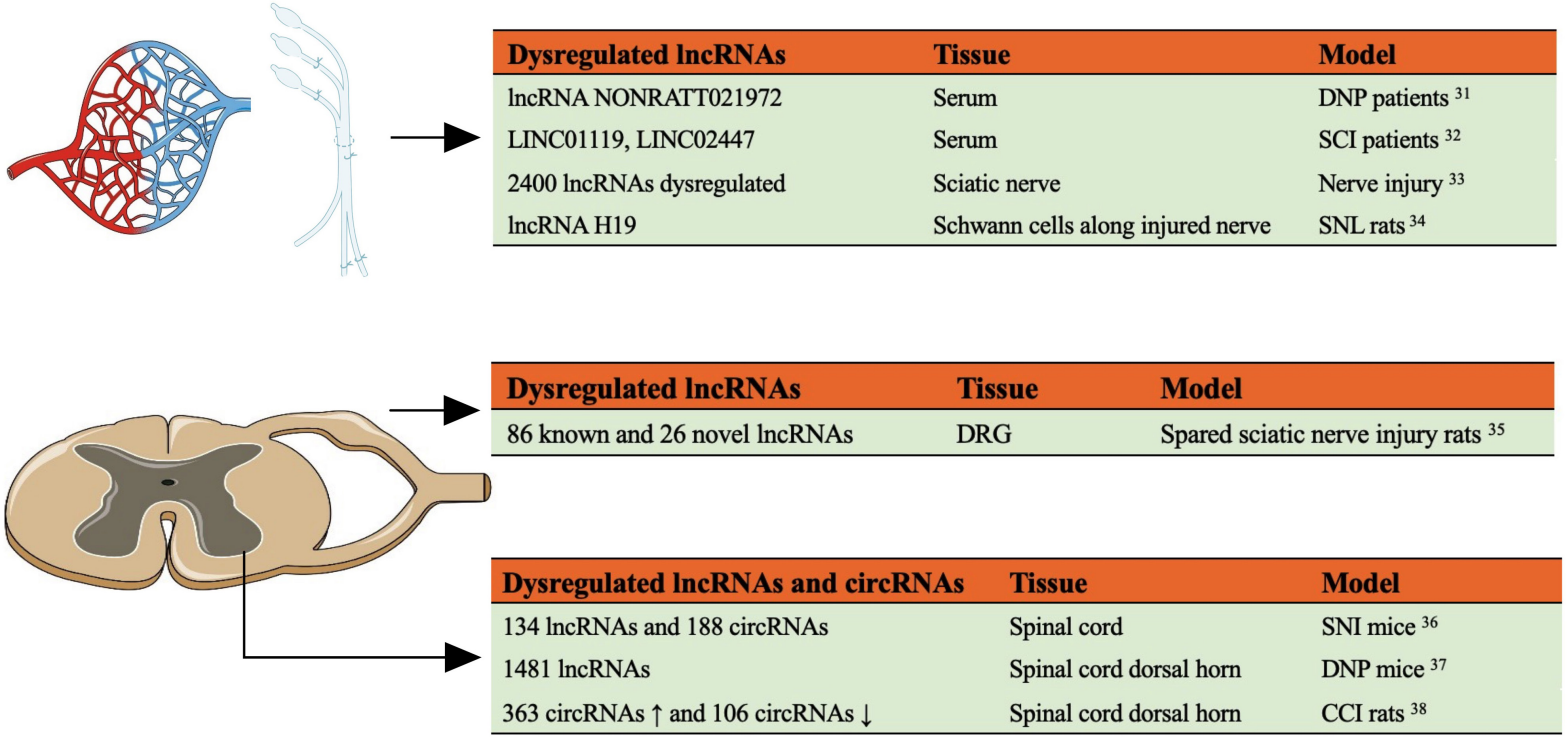
Long non‐coding RNA (lncRNAs) and circular non‐coding RNAs (circRNAs) expression change following peripheral nerve injury or noxious stimuli DNP, diabetic neuropathic pain; SCI, spinal cord injury; SNL, spinal nerve ligation; DRG, dorsal root ganglion; SNI, spared nerve injury; CCI, chronic constriction injury

circRNAs have characteristic circularized structures following the backsplicing of exons from antisense RNAs (AS RNAs) or mRNAs and, thus, are highly stable. Most circRNAs are highly conserved among species and lack translation potential, despite cap‐independent translation. A recent study showed that 363 and 106 circRNAs were significantly dysregulated in the ipsilateral dorsal horn after nerve injury.^38^ (Figure 2).

## 4 STUDYING miRNA NEUROPATHIC PAIN MECHANISMS

miRNAs contribute to the development of neuropathic pain via diverse mechanisms, such as neuroinflammation, autophagy, abnormal ion channel expression, regulating pain‐related mediators, protein kinases, structural proteins, neurotransmission excitatory–inhibitory imbalances, and exosome miRNA‐mediated neuron–glia communication (Table 1).

**TABLE 1:** miRNAs expression change for the development of neuropathic pain

Mechanism	miRNA	Target	Tissue	Model
Neuroinflammation	miR‐101 ↓	NF‐κB ↑	Serum and sural nerve	NP patients^39^
	miR‐124, −155 ↑	SIRT1 ↓	Serum	NP patients^40^
	miR‐7a ↓	NEFL/STAT3 ↑	DRG	SNL rats^41^
	miR‐590‐3p ↓	RAP1A ↑	DRG	Diabetic NP mice^42^
	miR‐140 ↓	S1PR1 ↑	DRG	CCI rats^43^
	miR‐144 ↓	RASA1 ↑	DRG	CCI mice^44^
	miR‐130a‐3p ↓	IGF‐1/IGF‐1R ↑	Spinal cord	SCI rats^45^
	miR‐378 ↓	EZH2 ↑	Spinal cord	CCI rats^46^
	miR‐138 ↓	TLR4/MIP‐1α/CCR1 ↑	Spinal cord	pSNL rats^47^
	miR‐15a/16 ↑	GRK2 ↓ and p38 MAPK/NF‐κB ↑	Spinal cord	CCI mice^48^
	miR‐124 ↓	GRK2 ↓	Spinal cord microglia	SNI mice^49^
	miR‐214‐3p ↓	DNMT3a/CSF1/IL‐6 ↑	Spinal cord astrocytes	SNL rats^50^
	miR‐214 ↓	Nav1.3/Bax ↑	Spinal cord	SCI rats^51^
	miR‐128 ↓	p38 ↑	Spinal cord	SCI rats^52^
	miR‐128‐3p ↓	ZEB1 ↑	Spinal cord	CCI rats^53^
	miR‐200b, −429 ↓	ZEB1 ↑	Spinal cord	CCI rats^54^
	miR‐23a ↓	CXCR4/TXNIP/NLRP3 ↑	Spinal cord	pSNL mice^55^
	miR‐155 ↓	NOX2/ROS ↑	Spinal cord microglia/macrophages	SCI mice^56^
		TNFR1/p38‐MAPK/JNK/TRPA1 ↑	Spinal cord dorsal horn	Chemotherapy induced NP rats^58^
	miR‐155 ↑	SOCS1 ↓ and NF‐κB/p38 MAPK ↑	Spinal cord	CCI rats^61^
		Oxidative stress–TRPA1 pathway ↑	Spinal cord	Chemotherapy induced NP rats^62^
Autophagy	miR‐15a ↓	AKT3 ↑	Spinal cord microglia	CCI rats^63^
	miR‐145 ↓	AKT3 ↑	DRG	CCI rats^64^
	miR‐20b‐5p ↓	AKT3 ↑	Dorsal spinal cord	CCI rats^65^
	miR‐195 ↑	ATG14 ↓	Spinal cord microglia	SNL rats^66^
Ion channel expression	miR‐7a ↓	β2 subunit of Scn2b ↑	DRG	SNL and CCI rats^67^

miR‐30b ↓	Scn3a (encoding Nav1.3) ↑	DRG and spinal cord	SNL rats^68^

miR‐96 ↓	Scn3a (encoding Nav1.3) ↑	DRG	CCI rats^69^

miR‐384‐5p ↓	Scn3a (encoding Nav1.3) ↑	DRG and spinal cord	CCI rats^70^

miR‐30b‐5p ↓	Scn8a (encoding Nav1.6) ↑	DRG	Chemotherapy induced NP rats^71^

miR‐182 ↓	Scn9a (encoding Nav1.7) ↑	DRG	SNI rats^72^

miR‐30b ↓	Scn9a (encoding Nav1.7) ↑	DRG	SNI rats^73^

miR‐17‐92 cluster ↑	K_v_1.1, K_v_1.4, K_v_3.4, K_v_4.3, K_v_7.5, DPP10, Na_v_β1 ↓	DRG	SNL rats^74^

miR‐137 ↑	Kcna2 (encoding K_v_1.2) ↓	DRG and spinal dorsal horn	CCI rats^75^

miR‐183‐5p ↓	TREK‐1 ↑	DRG	CCI rats^76^

miR‐183 cluster ↓	Cacna2d1/Cacna2d2 ↑	DRG	SNL mice^77^

miR‐103 ↓	Cacna1c/Cacna2d1/Cacnb1 ↑	Spinal dorsal horn	SNL rats^78^

miR‐141‐5p ↓	TRPA1 ↑	DRG	Chemotherapy induced NP rats^80^

miR‐449a ↓	TRPA1/KCNMA1 ↑	DRG	SNI mice^81^
Dysregulation of pain‐related mediators, protein kinases, and structural proteins	miR‐455‐3p ↑	NGF and related genes ↓	Serum	HIV/AIDS patients with symptomatic distal sensory polyneuropathy and NP^82^
	miR‐1 ↓	BDNF ↑	Sciatic nerve	CCI rats^83^
	miR‐183 ↓	BDNF ↑	DRG	SNL rats^84^
	miR‐206 ↓	BDNF ↑	DRG	CCI rats^85^
	miR‐19a, −301, −132 ↓	MeCP2/BDNF ↑	DRG	SNI mice^86^
	miR‐30c‐5p ↑	TGF‐β ↓	Serum, CSF, DRG, and spinal cord	SNI rats^16^
	miR‐133a‐3p ↑	p‐p38 MAPK ↑	Sciatic nerve	DNP rats^87^
	miR‐132 ↓	MeCP2/p‐CREB ↑	Spinal cord	SNI mice^88^
	miR‐200b, −429 ↓	DNMT3a ↑	Nucleus accumbens neurons	SNL mice^89^
	miR‐1 ↓	Cx43 ↑	Sciatic nerve	CCI rats^83^
	miR‐15b ↑	BACE1 ↓	DRG	Chemotherapy induced NP rats^94^
Excitatory‐inhibitory imbalance	miR‐500 ↑	GAD67 ↓	Spinal cord dorsal horn	Chemotherapy induced NP or L5 VTR^95^
	miR‐23b ↓	Nox4 ↑ and GAD65/67 ↓	Spinal cord	SCI mice^96^
	miR‐539 ↓	NR2B ↑	Contralateral ACC	CCI rats^97^
EV miRNA‐mediated neuron–glia communication	miR‐21 ↑	Phagocyted by macrophages to promote a pro‐inflammatory phenotype	Serum exosomes	pSNL mice^98^
	miR‐21‐5p ↑	Phagocyted by macrophages to promote a pro‐inflammatory phenotype	DRG exosomes	SNI mice^99^
	miR‐23a ↑	Phagocyted by macrophages to target A20 and enhance MI polarization	DRG EVs	SNI mice^102^

Abbreviations: ACC, anterior cingulate cortex; BACE1, beta‐site amyloid precursor protein‐cleaving enzyme 1; BDNF, brain‐derived neurotrophic factor; CCI, chronic constriction injury; CCR1, C–C chemokine receptor 1; CSF, cerebro spinal fluid; CSF1, colony‐stimulating factor‐1; CXCR4, chemokine CXC receptor 4; DNMT3a, DNA methyltransferase 3a; DNP, diabetic neuropathic pain; DRG, dorsal root ganglion; EV, extracellular vesicle; EZH2, enhancer of zeste homolog 2; GRK2, G protein‐coupled receptor kinases 2; IGF‐1/IGF‐1R, insulin‐like growth factor‐1/insulin‐like growth factor‐1 receptor; KCNMA1, calcium‐activated potassium channel subunit α‐1; MeCP2, methyl cytosine–guanine dinucleotide (CpG)‐binding protein 2; MIP‐1a, macrophage inflammatory protein‐1 alpha; NEFL, neurofilament light polypeptide; NF‐κB, nuclear factor – kappa B; NGF, nerve growth factor; NLRP3, NOD‐like receptor protein 3; NOX2, NADPH oxidase 2; NP, neuropathic pain; pSNL, partial sciatic nerve ligation; RAP1A, Ras‐related protein 1A; RASA1, RAS P21 protein activator 1; ROS, reactive oxygen species; S1PR1, sphingosine‐1‐phosphate receptor 1; SCI, spinal cord injury; Scn2b, β2 subunit of the voltage‐gated sodium channel; SIRT1, histone deacetylase sirtuin 1; SNL, spinal nerve ligation; SOCS1, suppressor of cytokine signaling 1; STAT3, signal transducer and activator of transcription 3; TLR4, toll‐like receptor 4; TNFR1, TNF receptor 1; TRPA1, transient receptor potential ankyrin 1; TXNIP, thioredoxin‐interacting protein; VTR, ventral root transection; ZEB1, zinc finger E‐box binding homeobox 1.

### 4.1 miRNAs regulate neuroinflammation in neuropathic pain development

miRNA‐based epigenetic regulation is essential in neuroinflammation. miRNAs are predicted to regulate diverse neuroinflammation‐related targets along the pain processing pathways.

Among patients with neuropathic pain, miR‐101 decreased in the serum and sural nerve, which is related to nuclear factor‐kappa B (NF‐κB) signaling activation.^39^ Meanwhile, serum miR‐124a and miR‐155 expression was upregulated. They were identified to inhibit histone deacetylase sirtuin 1 (SIRT1) in primary human cluster of differentiation 4 (CD4) (+) cells and induce their differentiation toward regulatory T cells (Tregs), thus reducing pain‐related inflammation.^40^ Such miRNA–target interactions may act as an endogenous protective mechanism for neuropathic pain.

miR‐7a, expressed in small‐sized nociceptive DRG neurons, is downregulated after nerve injury, as reported, targeting neurofilament light polypeptides (NEFLs).^41^ NEFL encodes a neuronal protein vital for neurofilament formation and increases signal transducer and activator of transcription 3 (STAT3) phosphorylation, which is highly related to cell differentiation and neuroinflammation. In diabetic peripheral neuropathic mice, miR‐590‐3p was downregulated to disinhibit Ras‐related protein 1A (RAP1A) in the DRG tissue and inhibit neural T cells infiltration.^42^ Thus, exogenous miR‐590‐3p may be a potential alternative for neuropathic pain treatment. CCI downregulated miR‐140 and miR‐144 expression in the DRG. Intrathecally injected miR‐140 and miR‐144 agomir decreased inflammatory factor secretion and ameliorated hyperalgesia by targeting sphingosine‐1‐phosphate receptor 1 (S1PR1) and RAS P21 protein activator 1 (RASA1), respectively.^43^, ^44^

miRNAs are essential in neuropathic pain development via neuroinflammation‐related mechanisms in the spinal cord. miR‐130a‐3p targets and downregulates insulin‐like growth factor‐1/insulin‐like growth factor‐1 receptor (IGF‐1/IGF‐1R) expression to alleviate SCI‐induced neuropathic pain by mitigating microglial activation and NF‐κB phosphorylation.^45^ miR‐378 was decreased in CCI rats, inhibiting neuropathic pain development by targeting the enhancer of zeste homolog 2 (EZH2).^46^ EZH2 promotes neuropathic pain by increasing tumor necrosis factor‐alpha (TNF‐α), interleukin (IL)‐1β, and monocyte chemoattractant protein‐1 (MCP‐1) production. Intrathecally injecting miRNA‐138 lentivirus can remarkably alleviate neuropathic pain in partial sciatic nerve ligation (pSNL) rats by suppressing toll‐like receptor 4 (TLR4) and macrophage inflammatory protein‐1 alpha (MIP‐1α)/C‐C chemokine receptor 1 (CCR1) signaling pathways.^47^

miR‐15a/16 targets and downregulates G protein‐coupled receptor kinases 2 (GRK2) to disinhibit p38‐MAPK (mitogen‐activated protein kinase) and NF‐κB, contributing to neuroinflammation after CCI.^48^ GRK2‐deficient mice present a pro‐inflammatory phenotype in spinal cord microglia/macrophages, restored by miR‐124.^49^ SNL increased DNA methyltransferase 3a (DNMT3a) expression related to hypermethylation of the miR‐214‐3p promoter, resulting in miR‐214‐3p expression reduction, which enhanced astrocyte reactivity, colony‐stimulating factor‐1 (CSF1), and interleukin 6 (IL‐6) production, and hyperalgesia in rats.^50^ Electro‐acupuncture attenuated SCI by inhibiting Nav1.3 and Bax in the injured spinal cord through miR‐214 upregulation.^51^ Downregulated miR‐128 was reported to contribute to neuropathic pain via p38 or zinc finger E‐box binding homeobox 1 (ZEB1) activation in the spinal cord.^52^, ^53^ Meanwhile, ZEB1 was also targeted by miR‐200b/miR‐429, orchestrating neuropathic pain development.^54^

In a pSNL mouse model, nerve injury significantly reduced miR‐23a expression in spinal glial cells, concomitant with the upregulation of its target chemokine, CXC receptor 4 (CXCR4). In naïve mice, either miR‐23a downregulation or CXCR4 upregulation could active the thioredoxin‐interacting protein (TXNIP)/NOD‐like receptor protein 3 (NLRP3) inflammasome axis. Both intrathecal miR‐23a mimics and spinal CXCR4 downregulation by a lentivirus inhibited TXNIP or NLRP3 upregulation to alleviate hyperalgesia.^55^

Notably, one miRNA was shown to have distinctive targets in different animal models of the spinal cord. For example, miR‐155 downregulation targeted and upregulated NADPH oxidase 2 (NOX2) expression to induce reactive oxygen species (ROS) production after SCI, presenting a pro‐inflammatory phenotype in microglia/macrophages.^56^ The ability to induce glial polarization was also observed in cultured BV‐2 microglia.^57^ In bortezomib‐induced neuropathic pain rats, downregulated miR‐155 upregulated TNF receptor 1 (TNFR1) expression, which activated its downstream signaling pathways, including p38‐MAPK, c‐Jun N‐terminal kinase (JNK), and transient receptor potential ankyrin 1 (TRPA1).^58^ Therefore, it is suggested that miR‐155 might act as an intervention target for neuropathic pain. As expected, treatment with ibuprofen and L‐arginine delayed the behavioral pain changes while inhibiting spinal miR‐155 and NO.^59^ miR‐155‐5p is also known to destabilize the blood–nerve barrier and expression of tight junction proteins, such as claudin‐1 and zonula occludens‐1 (ZO‐1). Tissue plasminogen activator (tPA) could transiently open such barriers to facilitate topically applying analgesics, via miR‐155‐5p upregulation.^60^ However, we also noted that CCI upregulated but did not downregulate spinal cord miR‐155, and miR‐155 inhibition enhanced suppressor of cytokine signaling 1 (SOCS1) expression to inactivate inflammation via NF‐κB and p38‐MAPK inhibition.^61^ In oxaliplatin‐induced peripheral neuropathic pain, spinal cord miR‐155 expression was also upregulated, and intrathecally injecting the miR‐155 inhibitor attenuated hyperalgesia in rats, possibly by inhibiting oxidative stress–TRPA1 pathways.^62^ The underlying mechanisms of such distinctive miR‐155 expression change are still unknown and require further research into different pain models.

### 4.2 Autophagy

miRNA‐related autophagy is involved in neuropathic pain regulation. As reported, miR‐15a downregulated and stimulated AKT serine/threonine kinase 3 (AKT3), and inhibited autophagy post‐CCI.^63^ Impaired autophagy participates in neuropathic pain development. Intrathecal miR‐15a agomir prominently suppressed AKT3 expression, induced autophagy, and attenuated CCI‐induced neuropathic pain. Similar to miR‐15a, miR‐145 and miR‐20b‐5p contributed to neuropathic pain regulation via protein kinase B (AKT)‐related autophagy pathways.^64^, ^65^ Meanwhile, miR‐195 in the spinal cord was observed to upregulate post‐SNL, targeting and inhibiting Autophagy‐Related 14 (ATG14) and its autophagy activation.^66^ The miR‐195 inhibitor activated autophagy and suppressed neuroinflammation in vivo and in vitro.

### 4.3 The contribution of miRNAs to ion channel expression in neuropathic pain

Another class of miRNA‐based regulation focuses on ion channels, including sodium, potassium, and calcium channels, and transient receptor potential (TRP) channels, to modulate action potential production, firing rate, and neurotransmitter release.

Theoretically, miRNAs can simultaneously have multiple target mRNAs because precise matching is not a prerequisite for inhibiting the target sequence. For example, other than NEFL,^41^ miR‐7a targets the β2 subunit of the voltage‐gated sodium channel (*Scn2b*) with post‐transcriptional regulation to induce nociceptive DRG neurons hyperexcitability.^67^ Other than miR‐7a, the other known miRNA‐ targets involved in sodium channels expression include miR‐30b, ‐96, ‐384‐5p and their target *Scn3a* (encoding Na_v_1.3),^68^, ^69^, ^70^ miR‐30b‐5p and its target *Scn8a* (encoding Na_v_1.6),^71^ and miR‐182 and ‐30b and their target *Scn9a* (encoding Na_v_1.7).^72^, ^73^

A miRNA cluster is a polycistronic gene containing several miRNAs derived from a single primary or nascent transcript. Approximately 40% of miRNAs are predicted to form clusters, but their significance is still unknown. miR‐17‐92 is a miRNA cluster that includes six different members, of which miR‐18a, ‐19a, ‐19b, and ‐92a upregulation induces allodynia. The predicted targets of the miR‐17‐92 cluster encompass genes encoding diverse voltage‐gated potassium channels and their regulatory subunits, including K_v_1.1, K_v_1.4, K_v_3.4, K_v_4.3, K_v_7.5, dipeptidyl peptidase 10 (DPP10), and Na_v_β1.^74^ CCI upregulated miR‐137 to target and downregulate *Kcna2*, which encodes K_v_1.2 in the DRG and spinal dorsal horn.^75^ By contrast, CCI decreased miR‐183‐5p expression in the DRG, and the predicted target gene TREK‐1, a subunit of the 2P‐domain K^+^ channel, was increased.^76^

Furthermore, the miRNA‐183 cluster (miR‐183, part of miR‐96/182/183) regulates either basal mechanical or neuropathic pain.^77^ The miR‐183 cluster targets *Cacna2d1* and *Cacna2d2*, which encode auxiliary voltage‐gated calcium channel subunits α2δ‐1 and α2δ‐2, to affect nociceptor excitability. Nerve injury downregulated miR‐103 in the spinal cord, which simultaneously targeted and inhibited *Cacna1c*, *Cacna2d1,* and *Cacnb1* and encoded the Cav1.2‐α1, α2δ1, and β1 subunits of the voltage‐gated calcium channels macromolecular complex Cav1.2 L‐type calcium channel, respectively.^78^ Intrathecal miR‐103 successfully relieved neuropathic pain.

In addition to ion channels, TRP channels are ligand‐gated ion channels that promote painful sensations.^79^ In the DRG, TRPA1 participates in miR‐141‐5p alleviated oxaliplatin‐induced neuropathic pain.^80^ In SNI mice, miR‐449a ameliorated neuropathic pain by decreasing the activity of TRPA1 and calcium‐activated potassium channel subunit α‐1 (KCNMA1), thus acting as a potential therapeutic alternative for treating neuropathic pain.^81^

### 4.4 miRNAs regulate pain‐related mediators, protein kinases, and structural proteins

miRNAs regulate diverse pain‐related mediators, protein kinases, and structural proteins along the pain processing pathways. For HIV‐associated symptomatic distal sensory polyneuropathy and neuropathic pain, serum miR‐455‐3p acted as a potential biomarker, possibly targeting multiple genes involved in peripheral neuropathic pain, such as nerve growth factor (NGF) and related genes.^82^ Brain‐derived neurotrophic factor (BDNF), another well‐recognized pain mediator, is a common miR‐1, ‐183, and ‐206 target.^83^, ^84^, ^85^ After nerve injury, several miRNAs, including miR‐19a, ‐301, and ‐132, were downregulated, and their target methyl cytosine–guanine dinucleotide (CpG)‐binding protein 2 (MeCP2) expression was increased, leading to concomitant BDNF upregulation.^86^ miR‐30c‐5p was upregulated in the serum and CSF of patients with chronic peripheral ischemia.^16^ A miR‐30c‐5p inhibitor intraventricular injection postponed neuropathic pain development and fully reversed hyperalgesia in rodents. Transforming growth factor beta (TGF‐β) participates in the effects of miR‐30c‐5p, which refers to the endogenous opioid analgesic system.^16^

miRNAs dysregulate specific pain‐related protein kinases. For example, in DNP rats, miR‐133a‐3p was dysregulated in the sciatic nerve, interacting with p‐p38 MAPK to participate in the development of neuropathic pain.^87^ Via a methyl CpG‐binding domain and transcriptional repressor domain, MeCP2 acts as a transcriptional repressor, and its overexpression improves neuropathic pain, potentiating an anti‐nociceptive effect of MeCP2. The authors also noted that MeCP2 expression changed post‐transcriptionally, and its mRNA level did not significantly change after SNI. However, the protein level was upregulated. During the development period of neuropathic pain, phospho‐cAMP response element‐binding protein (p‐CREB) elevated rapidly but returned 3–7 days after SNI, concomitant with miR‐132 downregulation, which targets MeCP2 and inhibits its expression post‐transcriptionally.^88^ SNL decreased miR‐200b and miR‐429 in NAc neurons, along with an upregulation of their target (DNMT3a).^89^ Further mechanism studies found that DNMT3a in the NAc was expressed in NR1 immunoreactive neurons, suggesting the dysregulation of ‘mesolimbic motivation circuitry’ for neuropathic pain development.

The CCI induced time‐dependent miR‐1 downregulation in injured sciatic nerves. This change in expression was related to the upregulation and translocation of the miR‐1‐targeted connexin 43 (Cx43), the major connexin of astrocytes.^83^ miR‐1 mimics could reduce Cx43 expression in cultured human glioblastoma cells. However, miR‐1 mimics intraneural transfection failed to alter Cx43 protein expression and did not improve pain behavior. The authors attributed such treatment failure to insufficient inhibition of miR‐1 for Cx43 via intraneural injection. Alternatively, there were regulatory mechanisms for Cx43 in vivo other than miR‐1.^90^ However, miR‐1 in the DRG was DE according to peripheral nerve injury type. CCI, pSNL, and sural nerve injury downregulated miR‐1. However, axotomy of the sciatic nerve and tibial nerve injury increased its expression in the DRG.^83^, ^91^, ^92^ As previously described, miR‐1 was also upregulated following capsaicin treatment and bone cancer pain.^91^, ^93^ Additionally, miR‐1 downregulation inhibited bone cancer pain. Collectively, the action of miR‐1 on pain seems to be complex and stimulus‐dependent.

Notably, specific miRNAs can promote neuropathic pain development via diverse mechanisms. For example, beta‐site amyloid precursor protein‐cleaving enzyme 1 (BACE1), a membrane protease essential for myelination, was downregulated after miR‐15b overexpression in vitro or in the DRG of chemotherapy‐related neuropathic pain rats.^94^ BACE1‐mediated neuregulin 1 reduction decreases nerve conduction velocity. In addition, BACE1 modulated Na_v_β2 subunit expression and neuronal activity and regulated inflammation‐related TNFR expression.

### 4.5 Neurotransmission excitatory–inhibitory imbalances

Neurotransmission excitatory and inhibitory imbalances in the spinal cord also contribute to neuropathic pain development. For example, miR‐500 increased to regulate glutamic acid decarboxylase 67 (GAD67) expression and target the specific site of *Gad1* in the dorsal horn.^95^ GAD67 expression reduction inhibited the function of GABAergic neurons and the resultant inhibitory synaptic transmission dysregulation contributed to neuropathic pain development. In addition, miR‐23b is crucial for improving neuropathic pain in the injured spinal cord by downregulating its target gene, Nox4, which further normalizes glutamic acid decarboxylase 65/67 (GAD65/67) expression and protects GABAergic neurons from apoptosis.^96^ Microarray analysis showed that miR‐539 was prominently reduced in the contralateral anterior cingulate cortex (ACC) after CCI, which is related to enhanced NR2B protein expression. Injecting miR‐539 mimics into the contralateral ACC attenuated CCI‐evoked mechanical hyperalgesia, suggesting that the N‐methyl‐D‐aspartate (NMDA) receptor NR2B subunit regulates neuropathic pain.^97^

### 4.6 EV miRNAs‐mediated neuron–glia communication

We mainly focused on exosomal miR‐21 with regard to EV miRNAs in neuropathic pain. As aforementioned, SNL, DRT, or VRT could upregulate miR‐21 in the injured DRG.^22^ Notably, miR‐21 was also increased in serum exosomes from nerve‐ligated mice.^98^ In another in‐depth study, macrophages readily took up pure sensory neuron‐derived exosomes encompassing miR‐21‐5p to promote a pro‐inflammatory phenotype.^99^ Either intrathecal miR‐21‐5p antagomir or miR‐21 conditional deletion in sensory neurons ameliorated hyperalgesia and macrophage recruitment in the DRG.

Unlike the miRNA target gene inhibitory mechanism, miR‐21 acts as an endogenous toll‐like receptor 8 (TLR8) ligand, leading to neuropathic pain development.^100^ TLR8, a nucleic acid‐sensing receptor, is located in the endosomes and lysosomes, leading to ERK‐mediated inflammatory mediator production and neuronal activation after SNL. Although miR‐21 and TLR8 are only distributed in small‐ and medium‐sized neurons, miR‐21 can also be derived from large‐sized neurons and reach TLR8 in the endosomes of other types of neurons.^101^ Similar to miR‐21, DRG sensory neurons secreted miR‐23a‐enriched EVs following nerve injury and were taken up by macrophages to enhance M1 polarization in vitro. A20, an NF‐κB signaling pathway inhibitor, is a verified miR‐23a target gene.^102^ Moreover, intrathecally delivering EVs‐miR‐23a antagomir attenuated neuropathic hyperalgesia and reduced M1 macrophages by inhibiting A20 to activate NF‐κB signaling.

## 5 STUDYING lncRNAs AND circRNAs NEUROPATHIC PAIN MECHANISMS

lncRNAs and circRNAs are essential in neuropathic pain development by acting as AS RNA or miRNA sponges, epigenetically regulating pain‐related molecules expression, or modulating miRNA processing (Table 2).

**TABLE 2:** Long non‐coding RNAs (lncRNAs) and circular non‐coding RNAs (circRNAs) expression change for the development of neuropathic pain

Mechanism	lncRNA	Mechanism	Tissue	Model
lncRNAs act as AS RNA	Egr2‐AS‐RNA ↑	Egr2‐AS‐RNA inhibition delays peripheral myelination	Schwann cells	Sciatic nerve transection mice^103^
	Kcna2‐AS‐RNA ↑ ↑	Kcna2‐AS‐RNA targets Kcna2 mRNA and reduces Kv current	DRG	SNL rats^104^
	Scn9a NAT	NAT inhibits Scn9a mRNA and its encoding protein Na_v_1.7 and current	DRG	CCI mice^105^
lncRNAs act as miRNA sponges	XIST ↑	Sponge miRNA‐137, −150, −154‐3p, −544 to disinhibit corresponding targets expression including TNFAIP1, ZEB1, TLR5, and STAT3	Spinal cord	CCI rats^106^, ^107^, ^108^, ^109^
	NEAT1 ↑	Form NEAT1‐miR‐381‐HMGB1 axis to upregulate HMGB1 expression	Spinal cord	CCI rats^110^
		Form NEAT1‐miR‐128‐3p‐AQP4 axis to upregulate AQP4 expression	Spinal cord	SCI rats^111^
	circHIPK3 ↑	Form circHIPK3‐miR‐124 axis to promote neuroinflammation	DRG	NP rats^113^
	ciRS‐7 ↑	Form ciRS‐7‐miR‐135a‐5p axis to promote autophagy and inflammation	Spinal cord	CCI rats^114^
	cirZNF609 ↑	Form cirZNF609‐miR‐22‐3p‐ENO1 axis to promote neuroinflammation	Spinal cord	CCI rats^115^
	circ_0005075 ↑	Form circ_0005075‐miR‐151a‐3p‐NOTCH2 axis to promote neuroinflammation	Spinal cord	CCI rats^116^
lncRNAs regulate pain‐related molecules expression	DS‐lncRNA ↓	DS‐lncRNA downregulation upregulates G9a, as well as downregulates G9a‐controlled opioid receptors and Kcna2	DRG	SNL mice^117^
	uc.48+ ↑	uc.48+ increases P2X3 receptor expression and ERK1/2 activation	DRG	DNP rats^119^
	BC168687 ↑	BC168687 increases P2X7 receptor expression, SGCs activation, and serum NO	DRG	DNP rats^120^
	BC168687 ↑	BC168687 increases TRPV1 expression	DRG	DNP rats^121^
	NONARATT02197 ↑	NONARATT02197 activates TNF‐a related pathway	DRG	DNP rats^31^
	NONARATT02197 ↑	NONARATT02197 increases P2X receptor expression	DRG	DNP rats^122^
	NONARATT02197 ↑	NONARATT02197 increases P2X7 receptor expression	DRG	DNP rats^123^
	MRAK009713 ↑	MRAK009713 increases P2X3 receptor expression	DRG	CCI rats^124^
	Lncenc1 ↑	Lncenc1 interacts with EZH2 and downregulates Bai1 expression in microglia	DRG	pSNL mice^125^
	SNHG1 ↑	SNHG1 targets CDK4 to induce neuroinflammation	Spinal cord	SNL rats^126^
	PKIA‐AS1 ↑	PKIA‐AS1 targets CDK6 to induce neuroinflammation	Spinal cord	SNL rats^127^
	Kcna2‐AS‐RNA ↑	It promotes pSTAT3 nucleus translocation and spinal cord neuroinflammation	Spinal cord	Postherpetic neuralgia rats^129^
lncRNAs modulate miRNA processing	uc.153 ↑	uc.153 negatively modulates pre‐miR‐182‐5p processing and maturation, disinhibits EphB1 and p‐NR2B expression	Spinal cord	CCI mice^130^

Abbreviations: AQP4, aquaporin 4; AS RNA, antisense RNA; CCI, chronic constriction injury; CDK4, cyclin‐dependent kinase 4; CDK6, cyclin‐dependent kinase 6; DNP, diabetic neuropathic pain; DRG, dorsal root ganglion; ENO1, enolase 1; EphB1, ephrin type‐b 1; EZH2, enhancer of zeste homolog 2; HMGB1, high mobility group box 1; NAT, natural antisense transcript; NOTCH2, NOTCH receptor 2; NP, neuropathic pain; pSNL, partial sciatic nerve ligation; pSTAT3, phosphorylated signal transducer and activator of transcription 3; SCI, spinal cord injury; SGC, satellite glia cell; SNL, spinal nerve ligation; STAT3, signal transducer and activator of transcription 3; TLR5, toll‐like receptor 5; TNFAIP1, tumor necrosis factor alpha‐induced protein 1; TRPV1, transient receptor potential vanilloid 1; XIST, X‐inactive specific transcript; ZEB1, zinc finger E‐box binding homeobox 1.

### 5.1 lncRNAs act as AS RNA

After peripheral nerve injury, Egr2‐AS‐RNA is upregulated in Schwann cells.^103^ On the early growth response 2 (Egr2) promoter, Egr2‐AS‐RNA recruits an epigenetic silencing complex to downregulate Egr2, essential for peripheral myelination. Ectopic Egr2‐AS‐RNA expression in DRG cultures downregulates *Egr2* mRNA and induces demyelination. In vivo, Egr2‐AS‐RNA inhibition reverts *Egr2*‐related gene expression and delays demyelination.

Voltage‐gated potassium channel (Kv) Kcna2‐AS‐RNA is an endogenous, highly conserved, and widely explored lncRNA in neuropathic pain.^104^ It is a natural antisense transcript (NAT), distributed in the cytoplasm and targets *Kcna2* mRNA, which encodes the pain regulation‐related membrane K_v_1.2 subunit. Kcna2‐AS‐RNA was time‐dependently upregulated in the inflicted rat DRG after nerve injury. It decreased the total K_v_ current, upregulated DRG neurons excitability, and produced neuropathic pain symptoms.

The complementary strand of DNA opposite the Scn9a gene encodes Scn9a NAT, another antisense lncRNA expressed in the DRG.^105^ Scn9a NAT is suggested to be a negative regulator of *Scn9a* mRNA. Scn9a NAT overexpression inhibited *Scn9a* mRNA, its encoded protein Na_v_1.7, and Na_v_1.7 currents in DRG neurons. However, Scn9a NAT and *Scn9a* mRNA levels did not significantly change in the injured DRG until 2 weeks after nerve injury. More work is required to determine whether NAT can confer analgesia and reduce pain in the neuropathic pain state.

### 5.2 lncRNAs act as miRNA sponges

lncRNAs may act as miRNA sponges to form the lncRNA‐miRNA‐mRNA axis and regulate target gene expression. For example, the lncRNA X‐inactive specific transcript (XIST) is upregulated in the dorsal horn of the spinal cord after CCI. By sponging miR‐137, ‐150, ‐154‐5p, and ‐544, XIST can inhibit the expression of corresponding targets, including TNF‐α‐induced protein 1 (TNFAIP1), ZEB1, TLR5, and STAT3.^106^, ^107^, ^108^, ^109^ TNFAIP1 activates the NF‐κB signaling pathway, while ZEB1, TLR5, and STAT3 are crucial in the neuroinflammatory response. XIST inhibition markedly ameliorates neuropathic pain development. In addition, lncRNA nuclear enriched abundant transcript 1 (NEAT1) was upregulated in the spinal cord dorsal horn to form NEAT1‐miR‐381‐high mobility group box 1 (HMGB1) and NEAT1–miR‐128‐3p–aquaporin 4 (AQP4) axes following CCI and SCI, respectively.^110^, ^111^ NEAT1 downregulation inhibited IL‐6, IL‐1β, and TNF‐α to improve neuropathic pain.

Although the function of circRNAs as miRNA sponges is still largely unknown, they have been termed as competing endogenous RNAs that bind to target miRNAs and regulate their function.^112^ For example, circHIPK3 is a circRNA highly enriched in serum from DNP patients and in the DRG from DNP rats.^113^ circHIPK3 sponges miR‐124 to promote neuroinflammation, pointing to the involvement of the circHIPK3–miR‐124 axis during DNP. circRNA ciRS‐7 participates in neuropathic pain progression by sponging miR‐135a‐5p to regulate autophagy and inflammation in the spinal cord of CCI rats.^114^ Besides, cirZNF609–miR‐22‐3p‐enolase 1 (ENO1) and circ_0005075–miR‐151a‐3p‐NOTCH receptor 2 (NOTCH2) regulatory axes upregulate inflammatory factor expression and promote neuropathic pain development in the spinal cord after CCI.^115^, ^116^

### 5.3 lncRNAs epigenetically regulate pain‐related molecules expression

In addition to acting as a miRNA sponge, lncRNAs can epigenetically regulate the expression of pain‐related molecules. For example, peripheral nerve injury decreases the expression of DRG‐specifically enriched lncRNAs (DS‐lncRNAs) in the injured DRG. Restoring DS‐lncRNAs blocks nerve injury‐induced increases in euchromatic histone lysine N‐methyltransferase 2 (Ehmt2) mRNA and its encoding protein G9a, reverses G9a‐related decreases in opioid receptors and Kcna2 in the injured DRG, and ameliorates nerve injury‐induced pain hypersensitivity.^117^ In addition, transcriptome screening in the DRG of DNP rats^118^ has identified diverse dysregulated lncRNAs, including uc.48+,^119^ BC168687,^120^, ^121^ NONRATT021972,^31^, ^122^, ^123^ MRAK009713,^124^ and Lncenc1.^125^ The expression of uc.48+, BC168687, NONRATT021972, MRAK009713, and Lncenc1 was prominently higher in the DRG of neuropathic pain rats.^119^, ^120^, ^121^, ^122^, ^123^, ^124^, ^125^ Blocking the upregulation via intrathecal or intravenous administration of the corresponding small‐interfering RNA (siRNA) may alleviate neuropathic pain by inhibiting the excitatory transmission mediated by purinergic receptors,^119^, ^120^, ^122^, ^123^, ^124^ TNF‐α‐related pathways,^31^ transient receptor potential vanilloid 1 (TRPV1),^121^ or EZH2.^125^

As demonstrated by a luciferase assay and RNA‐binding protein immunoprecipitation, lncRNA small nucleolar RNA host gene 1 (SNHG1) can induce neuropathic pain in the spinal cord by binding to the promoter region of cyclin‐dependent kinase 4 (CDK4), stimulating its expression.^126^ SNHG1 knockdown alleviated neuropathic pain development, and SNHG1 overexpression was able to induce neuropathic pain. Similarly, lncRNA PKIA‐AS1 participates in SNL‐induced neuropathic pain by downregulating DNA methyltransferase 1‐catalyzed cyclin‐dependent kinase 6 (DNMT1‐catalyzed) CDK6 promoter methylation and regulating CDK6.^127^ Cyclin‐dependent kinases (CDKs) transcriptionally enhanced pro‐inflammatory gene expression during the G1 cell phase. Furthermore, CDK6 recruitment to the nuclear chromatin fraction by cytokines is related to NF‐κB, STAT, and activator protein 1 (AP‐1) activation to induce neuroinflammation.^128^ Other than the DRG, lncRNA Kcna2‐AS‐RNA was also highly expressed in the spinal cord of postherpetic neuralgia rats, and its downregulation alleviated neuropathic pain by reducing phospho‐STAT3 (pSTAT3) translocation from the cytoplasm to the nucleus and then inhibiting spinal astrocytes activation.^129^

### 5.4 lncRNAs modulate miRNA processing

Specific lncRNAs are essential in neuropathic pain by modulating miRNA processing. For example, the transcribed ultraconserved lncRNA uc.153 was prominently increased in the spinal cord of rats with CCI neuropathic pain. uc.153 knockdown reversed CCI‐induced pain behaviors and spinal neuronal hypersensitivity. Mechanistically, uc.153 negatively modulated Dicer‐mediated pre‐miR‐182‐5p processing and inhibited maturation. Meanwhile, spinal miR‐182‐5p downregulation increased the expression of its target, ephrin type‐b (EphB1), and p‐NR2B (phosphorylated N‐methyl‐D‐aspartate receptor (NMDAR) 2B subunit) expression and facilitated hyperalgesia.^130^

Collectively, lncRNAs are crucial in neuropathic pain via diverse mechanisms. Notably, one lncRNA may act with more than one mechanism to regulate target gene expression. For example, SNL upregulated circAnks1a in the cytoplasm and nucleus. circAnks1a enhances the interplay between Y‐box‐binding protein 1 (YBX1) and transportin‐1 to facilitate YBX1 nuclear translocation in the cytoplasm. Meanwhile, in the nucleus, circAnks1a combines with the *Vegfb* promoter and recruits YBX1 to the *Vegfb* promoter to enhance its transcription. Additionally, cytoplasmic circAnks1a sponges miR‐324‐3p to regulate vascular endothelial growth factor B (VEGFB) expression. VEGFB binding to its receptor results in the activation of various downstream targets, including p38‐MAPK, PKB/AKT (protein kinase B), extracellular signal‐regulated kinase (ERK)/MAPK, and phosphoinositide 3‐kinase (PI3K).^131^ Therefore, VEGFB upregulation increases dorsal horn neuron excitability and contributes to pain hypersensitivity after nerve injury.^132^

We also noted that specific lncRNAs were DE following various nerve injuries. For example, lncRNA Malat1 was upregulated after CCI to sponge miR‐206, and Malat1 suppression delayed neuropathic pain progression via miR‐206‐ZEB2 axis‐mediated neuroinflammation inhibition.^133^ Conversely, in rats with complete brachial plexus avulsion‐induced neuropathic pain, Malat1 decreased in spinal cord neurons, and such downregulation increased neuronal spontaneous electrical activity via calcium flux regulation.^134^ The reason for such distinctive Malat1 expression following different pain models is still unclear, and further research is required.

## 6 CHALLENGES AND CONSIDERATIONS IN DELIVERING miRNA‐AND lncRNAs‐BASED THERAPETUTICS

There are two main strategies for modulating miRNA function during pain treatment: upregulation or downregulation of specific miRNAs. miRNA mimics or virus‐based constructs were used to upregulate miRNA expression. miRNA inhibitors, miRNA sponges, or inhibition of a particular miRNA‐mRNA interaction has been applied to downregulate specific miRNAs. The design of lncRNA‐based therapeutics includes diverse approaches, such as post‐transcriptional inhibition of lncRNAs by antisense oligonucleotides or siRNA and steric blockades of lncRNA–protein interactions by small molecules and morpholino.^135^

However, there is much work to be done before miRNA‐ and lncRNA‐based analgesics can be clinically used for various reasons, including their conservation among species, proper delivery, stability, off‐target effects, and potential activation of the immune system.

First, most available findings regarding the efficacy of miRNAs or lncRNAs during neuropathic pain are based on animal but not human studies. Whether such results can be extrapolated to humans and their translational potential is still unknown because of conservation among species.

Second, the blood–brain barrier (BBB) is a practical challenge for delivering RNA‐based therapeutics into the central nervous system (CNS) via intravenous injection. Viral vectors, polypeptides, aptamers, and particular chemical modifications have been developed. Supplementing cholesterol molecules to the sense strand of a miRNA mimic or inhibitor has proven to be an efficient strategy. Cholesterol‐conjugated siRNAs showed better silencing potency than unconjugated siRNAs and presented a high efficacy for delivery to oligodendrocytes in the CNS.^136^ Another proven method is the immunoliposome, a combination of liposomes, receptor‐targeted monoclonal antibodies, and the target molecules.^137^ An immunoliposome nanocomplex has been reported to deliver therapeutic nucleic acids across the BBB into the deep brain by transferrin receptors.^138^ In addition, intrathecal injections are a feasible approach in animal studies for neuropathic pain treatment. As reported, miR‐146a attenuated neuropathic pain partially by inhibiting TNF receptor‐associated factor 6 (TRAF6) and its downstream phospho‐JNK/C–C motif chemokine ligand 2 (pJNK/CCL2) signaling in the spinal cord.^139^ Intrathecal injection of miR‐146a‐5p encapsulated nanoparticles provided an analgesic effect via NF‐κB and p38‐MAPK inhibition in spinal microglia.^140^ In recent years, poly (D, L‐lactic‐co‐glycolic acid) (PLGA)‐nanoparticles have been applied to deliver siRNAs and plasmids into the spinal cord to treat neuropathic pain in rats.^141^ PLGA copolymer is a promising US Food and Drug Administration (FDA)‐approved gene transmission material because of its biodegradability and biocompatibility in humans.^142^ Intrathecal treatments with C‐X3‐C motif chemokine receptor 1 (CX3CR1), p38, p66shc siRNA encapsulated PLGA nanoparticles, or forkhead box P3 (Foxp3) plasmid‐encapsulated PLGA nanoparticles inhibit microglial activation and hyperalgesia in SNL rats.^141^, ^143^, ^144^, ^145^

Exosomes are another promising delivery carrier for treating neuropathic pain. Exosomes are natural membranous microvesicles that carry RNAs, with the advantage of being efficient, cell‐free, and nonimmunogenicity. Intravenous injecting neuron‐targeted exosomes delivered the carried siRNAs to neurons, microglia, and oligodendrocytes to knock down specific gene expression in mice brains.^146^ This approach enabled cell‐specific delivery of the siRNA‐cargo across the BBB. Mesenchymal stem cells (MSCs) are pluripotent stem cells with immunomodulatory, anti‐inflammatory, and nutritional properties. The treatment efficacy of exosomes derived from MSCs has been proven in neuropathic pain.^147^ Intrathecal, local, or subcutaneous application of exosomes obtained from human umbilical cord MSCs could mitigate nerve injury‐induced hyperalgesia.^148^, ^149^, ^150^ Furthermore, immunofluorescence results showed that most intrathecally injected exosomes could be found in injured peripheral axons, the DRG, and the spinal dorsal horn, potentiating a homing ability of exosomes.^148^

Third, although miRNAs are relatively stable in vivo, they have long‐lasting efficacy and a high resistance to nucleolytic degradation compared to mRNAs. Particular chemical modifications are still required to prolong its half‐life or increase its stability, for example, by generating locked‐nucleic acids (LNAs). LNA modifications increase the RNA affinity of antisense oligonucleotides, presenting an excellent miRNA inhibitory activity at a low dosage.^151^ Additionally, the LNA technique was used to synthesize highly stable aptamers.^152^

Fourth, the off‐target effect is another consideration. One miRNA may regulate multiple genes, and the off‐target potential of one miRNA may produce undesirable side effects.^153^, ^154^

Finally, activation of the immune system is a potential adverse event. Specific CpG motifs in oligonucleotides could trigger nonspecific immunological activity.^155^ Therefore, RNA‐based therapeutics might also produce specific immune reactions after producing antibodies against the oligonucleotides.

## 7 CONCLUSION

Recent human and animal studies have identified accumulating dysregulated miRNAs and lncRNAs in the serum or along pain processing pathways following peripheral nerve injury or noxious stimulation. Experimental studies have validated their essential role in neuropathic pain. miRNAs contribute to neuropathic pain development via neuroinflammation, autophagy, abnormal ion channel expression, regulating pain‐related mediators, protein kinases, structural proteins, neurotransmission excitatory–‐inhibitory imbalances, and exosome miRNA‐mediated neuron–glia communication. Meanwhile, lncRNAs and circRNAs are crucial in neuropathic pain development by acting as AS RNA and miRNA sponges, epigenetically regulating pain‐related molecules expression, or modulating miRNA processing. However, more work is required before miRNA‐ and lncRNA‐based analgesics can be clinically used for various reasons, including their conservation among species, proper delivery, stability, off‐target effects, and potential activation of the immune system.

## AUTHOR CONTRIBUTIONS

Ming Jiang: Investigation, Methodology. Yelong Wang: Manuscript preparation. Jing Wang: Figures and tables preparation. Shanwu Feng: Supervision, Funding acquisition. Xian Wang: Conceptualization, Funding acquisition.

## CONFLICT OF INTERESTS

The authors declare no competing interest in this review.

## Data Availability

Data sharing not applicable to this article as no datasets were generated or analyzed during the current study.
